# Prevalence and Risk Factors for Oropharyngeal, Esophageal, and Oral Candidiasis in HIV‐Positive Individuals: A Systematic Review and Meta‐Analysis

**DOI:** 10.1155/ipid/6445594

**Published:** 2026-07-29

**Authors:** Xue Ting Tan, Siti Hamidah binti Abdul Rahman, Lee Lee Low, Nur Hasnah binti Ma′amor

**Affiliations:** ^1^ Bacteriology Unit, Institute for Medical Research, National Institutes for Health, Ministry of Health Malaysia, Setia Alam, Selangor, Malaysia, moh.gov.my; ^2^ Department of Pathology, Hospital Sultan Abdul Halim, Ministry of Health Malaysia, Sungai Petani, Kedah, Malaysia, moh.gov.my; ^3^ Department of Medicine, Hospital Sultanah Bahiyah, Ministry of Health Malaysia, Alor Setar, Kedah, Malaysia, moh.gov.my; ^4^ Sector Evidence Based Healthcare, Management Office, National Institutes for Health, Ministry of Health Malaysia, Setia Alam, Selangor, Malaysia, moh.gov.my

**Keywords:** esophageal candidiasis, immunocompromised, mucosal candidiasis, oral candidiasis, oropharyngeal candidiasis, risk factors

## Abstract

**Background:**

Oral, oropharyngeal, and esophageal candidiasis are common forms of mucosal candidiasis (MC) in people living with human immunodeficiency virus/acquired immunodeficiency syndrome (PLWHA). Despite differences in infection sites, oral, oropharyngeal, and esophageal candidiasis share common pathophysiological mechanisms, including etiologic agents, host susceptibility, and antibiotic use. Identifying prevalence and risk factors associated with these types of candidiasis in this population enables clinicians to manage these infections across the affected mucosal sites effectively.

**Objective:**

To synthesize the global prevalence and identify the risk factors for oral, oropharyngeal, and esophageal candidiasis in PLWHA.

**Methods:**

We searched PubMed, Embase, Scopus, and Cochrane and extracted the prevalence data and adjusted odds ratios (ORs) for each risk factor associated with MC. We calculated pooled ORs for risk factors present in a minimum of two studies.

**Results:**

Fifteen studies were included. The overall prevalence of MC in PLWHA was 39.0% (95% CI: 27%–52%; *I* ^2^  = 99.74%, *p* = 0.001). However, male gender (pooled OR 1.53, 95% CI 1.12–2.09, *p* = 0.007), CD4 cell count below 200 cells/µL (pooled OR 2.41; 95% CI 1.36–4.29; *p* = 0.003), and lack of highly active antiretroviral therapy (HAART) treatment (pooled OR 1.76; 95% CI 1.16–2.66; *p* = 0.008) are significant risk factors for MC development in the PLWHA.

**Conclusions:**

The global overall prevalence of MC in PLHWA was 39%. Risk factors, including male gender, CD4 count, and HAART, are shown to have significant effects on the development of MC in PLWHA. The results highlighted the significance of promptly recognizing and addressing these risk factors to reduce the incidence of MC in PLWHA.

## 1. Introduction

Mucosal candidiasis (MC), as defined in this article, includes oral, oropharyngeal, and esophageal candidiasis, and is a common fungal infection in people living with human immunodeficiency virus/acquired immunodeficiency syndrome (PLWHA) [1, 2]. The prevalence of these infections varies, ranging from 59.3% to 90.0% for oropharyngeal candidiasis [3, 4], 25.1%–67.0% for oral candidiasis [5–7], and 9.8%–12.0% for esophageal candidiasis [8–10]. These infections happened due to several factors, including impaired mucosal barriers and reduced cellular immunity, especially CD4 T‐cell deficits [11, 12].

The common symptoms of these forms of MC include white, creamy lesions on the palate, inner cheeks, and tongue [13]. These lesions often cause pain and burning sensation, make swallowing difficult, cause loss of taste, and cause aversion to food [13]. It can result in restricted nutrition intake due to chronic pain or discomfort when chewing [14, 15]. Therefore, MC contributes significantly to morbidity in PLWHA [16] and serves as an indicator of underlying immune dysfunction, particularly esophageal candidiasis with increased risk of other severe infections [17].

This disease is mostly caused by *Candida* species which colonize mucous membranes [18, 19]. Although *Candida albicans* is the most common etiology, other *Candida* species, including *Candida tropicalis*, *Candida glabrata*, *Candida krusei*, and *Candida dubliniensis*, were also observed [20–22]. In PLWHA, systemic antifungals are often necessary to prevent relapse. Oral fluconazole or itraconazole is the preferred treatment, while echinocandins are reserved for severe MC refractory to azole therapy [23, 24].

Despite treatment, clinical outcomes vary, and recurrence is common [8, 25]. Prolonged or repeated exposure to antifungal therapy increases the risk of emerging resistance, highlighting the importance of identifying specific risk factors that contribute to the development and progression of MC [8, 25]. This study aimed to synthesize the prevalence and identify the risk factors for MC in PLWHA, to provide clinically relevant insights and improve the preventive and treatment strategies in this population.

## 2. Materials and Methods

### 2.1. Overview

We conducted a review according to a protocol registered in PROSPERO ID (CRD42024578816). The development of the protocol followed the guidelines from the Preferred Reporting Items for Systematic Review and Meta‐analysis Protocols (PRISMA‐P) 2015 statement [26]. The Preferred Reporting Items for Systematic Reviews and Meta‐Analyses (PRISMA) guidelines were used. We adopted the Cochrane Handbook for Systematic Review of Interventions for methodological recommendations [27].

### 2.2. Study Identification

An Internet search of relevant publications was performed using four databases: PubMed or MEDLINE, Cochrane Central Register of Controlled Trials (CENTRAL), Embase, and Scopus, from inception until June 06, 2024. The search terms used are listed in Appendix A (Supporting Table 1–4). All identified studies were extracted and managed using Mendeley Desktop (Version 1.19.8, 2024).

### 2.3. Eligibility Criteria

We included population‐observational studies comprising cohort, case–control, and cross‐sectional studies that investigate risk factors for MC in PLWHA as defined by the authors. The systematic review, meta‐analysis, literature review, or other types of review articles, letters to the editor, editorials, commentaries, or case studies were excluded. Both retrospective and prospective studies assessing prevalence and risk factors for the relevant forms of MC were included in this review. The control groups of the MC were investigated from the same population. The study inclusion was limited to full‐text English publications. For duplicate publications on the same cohorts, a single representative study was selected following consultation between authors. All relevant data were extracted from the included papers, regardless of the study period.

The studies were screened based on eligibility criteria defined according to PECOS: Population (P)—studies focusing on PLWHA; Exposure (E)—prevalence and risk factors associated with MC specifically referring to oral, oropharyngeal, and esophageal candidiasis; Comparison (C)—low to no risk factors for MC; and Outcome (O)—risk factors and prevalence of MC.

### 2.4. Search Methods for Identification of Studies

A comprehensive search strategy was developed using specific keywords to describe MC, PLWHA, and risk factors. A PubMed search strategy was developed and adapted for Embase, Scopus, and CENTRAL. All databases were searched from the date of inception to June 6, 2024. To identify additional published, unpublished, and ongoing trials, we screened the reference lists of relevant papers, using the Science Citation Index Cited Reference Search for forward tracking of relevant articles, and identified and searched relevant journals and conference proceedings. Additionally, reference lists were hand‐searched for relevant articles. The search strategy terms are listed in Appendix A.

### 2.5. Study Selection

Initially, all duplicates from the searches were removed from Mendeley Desktop, Version 1.19.8 (London, United Kingdom). Next, each title and abstract was independently screened by two reviewers (T.X.T. and S.H.B.A.R.). The third review author (L.L.L.) was consulted to make the final decision when a consensus could not be reached. All full‐text reports for inclusion were retrieved and then independently screened for study eligibility. Any discrepancies were resolved through discussion or consultation with the third review author (L.L.L.). The selection process is recorded in a PRISMA flow diagram [28].

### 2.6. Data Extraction

For each included study, two review authors (T.X.T. and S.H.B.A.R.) extracted data independently using a standardized electronic data collection form according to guidance from the Cochrane Handbook for Systematic Reviews of Interventions [29]. The selection process was documented, and the PRISMA flow diagram was generated [28]. For each study, we extracted the first author’s name, year, country where the study was performed, study design, prevalence, and risk factors using Microsoft Excel software.

### 2.7. Risk‐of‐Bias Assessment

Two review authors (T.X.T. and S.H.B.A.R.) have independently assessed the risk of bias for each included study. The Newcastle–Ottawa Scale (NOS) was applied for the study design: cross‐sectional, cohort, and case–control to assess the quality of the study [30]. A score of seven or more was classified as a “good quality,” while a score of less than seven was identified as “fair quality.” Any discrepancies were resolved by discussion with the third reviewer (L.L.L.), who served as an arbiter.

### 2.8. Data Synthesis and Analysis

Data were extracted from the included studies, and statistical analysis was performed using Stata software Version 17.0 [31] to calculate the pooled prevalence frequencies by applying random‐effects model [31, 32]. Heterogeneity was assessed using the *I* ^2^ statistic [33] to measure the variance due to study heterogeneity, with *I* ^2^ values of 25%, 50%, and 75% indicating low, moderate, and high heterogeneity, respectively [27].

Besides identifying the overall prevalence, we categorized the analysis according to the geographical region and types of candidiasis. Studies with comparable population exposures that provide sufficient data and outcomes, including mean, standard deviation (SD), odds ratios (ORs), and 95% confidence intervals (CIs), were included in the meta‐analysis. These analyses were conducted using Review Manager (RevMan) Version 5.4 to obtain the pooled ORs of risk factors for MC among PLWHA with random‐effects model. Results were presented as ORs with 95% CIs, while heterogeneity was assessed using *I* ^2^ statistic to determine the degree of variation. Additionally, a narrative synthesis was conducted to integrate evidence from studies reporting the risk factor but lacking sufficient data for meta‐analysis.

Next, the publication bias was evaluated using funnel plots and Egger’s test when at least 10 articles were available [34]. Furthermore, the sensitivity analysis was performed using leave‐one‐out analysis to evaluate the influence of individual study on the pooled prevalence estimate. Meta‐regression was performed using Stata Version 17.0 [31] if *I* ^2^ was higher (> 75%) to explore potential sources of heterogeneity across the studies. In this analysis, we also included the result for the correlation, SE, *z*‐value, *p*‐value, and 95% CI.

Furthermore, meta‐regression was performed to explore potential sources of between‐study heterogeneity when substantial heterogeneity was observed in the pooled prevalence analysis (*I* ^2^ > 75%). Meta‐regression was performed separately for the overall pooled prevalence of MC in PLWHA and for the pooled prevalence of oral candidiasis in PLWHA. Study‐level moderators were selected based on clinical and methodological relevance and the availability of extractable data across the included studies. The moderators assessed included sample size and geographical region. Sample size was selected to evaluate the possibility of small‐study effects, whereas geographical region was selected because differences in healthcare access, antiretroviral therapy availability, diagnostic practices, and public health strategies may contribute to variation in reported prevalence across studies. The overall significance of each moderator was assessed using the Wald *χ* ^2^ test. Meta‐regression results were reported as meta‐regression coefficients, standard errors (SE), *z*‐values, *p*‐values, and 95% CIs. Residual heterogeneity after meta‐regression was assessed using *τ* ^2^, *I* ^2^, and the residual Q statistic (Q_res). All analyses were conducted using Stata Version 17.0.

## 3. Results

### 3.1. Findings From the Search Strategies

**FIGURE 1:**
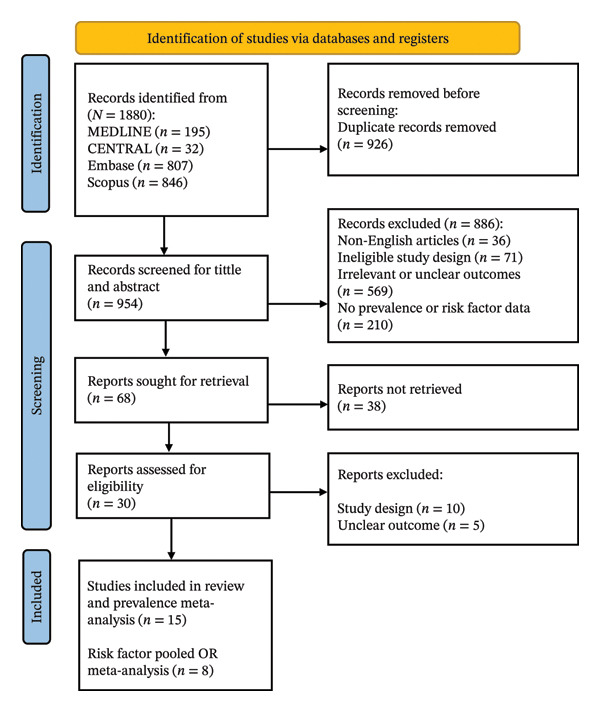
PRISMA flow diagram of study selection.

A total of 1880 records (195 records from MEDLINE, 807 from Embase, 846 from Scopus, and 32 from CENTRAL) were identified. Duplicate records were identified and excluded (*n* = 926), leaving a total of 954 articles. Following title and abstract screening and eligibility assessment, 15 articles were identified for full‐text review, with eight studies included in the meta‐analysis (Figure 1).

### 3.2. Description of the Included Studies

The characteristics of the 15 included studies are summarized in Table 1. All included studies contributed to the overall pooled prevalence analysis. However, only eight studies provided sufficient relevant data and were included in the pooled OR analysis of risk factors.

**TABLE 1:** Characteristics of the included studies.

No.	Study ID	Year	Country	Study design	Gender (male/female), *n*	Total IC patients, *n*	Number of MC in IC, *n*	Type of MC	*Candida* species
1	[20]	2018	Cameroon	Cross‐sectional	46/116	378	162	Oral	*C. albicans* *C. glabrata* *C. krusei* *C. tropicalis* *C. parapsilosis* *C. pseudotropicalis*

2	[35]	1993–1995	United States	Cohort	NA/867	867	103	Oral	NA

3	[21]	2012	Brazil	Cross‐sectional	84/63	147	89	Oral	*C. albicans* *C. parapsilosis* *C. tropicalis* *C. glabrata*

4	[40]	1996–2011	Brazil	Cross‐sectional	276/NA	534	267	Oral	NA

5	[43]	1998–2008	India	Cross‐sectional	2637/1087	3724	609	Oral	NA

6	[22]	2009	Taiwan, China	Cross‐sectional	99/6	105	54	Oropharyngeal	*C. albicans* *C. tropicalis* *C. glabrata* *C. dubliniensis* *C. parapsilosis* *C. krusei* *C. utilis* *C. guilliermondii* *C. ciferri*

7	[38]	2023	Iran	Case–control	70/34	104	100	Oral	NA

8	[37]	2016–2019	Indonesia	Case–control	151/56	448	207	Oral	NA

9	[41]	NA	Brazil	Cross‐sectional	53/26	79	16	Oral	NA

10	[44]	2018–2019	Iran	Cross‐sectional	115/86	201	88	Oral	*C. albicans* *C. dubliniensis* *C. glabrata* *C. tropicalis* *C. parapsilosis* *C. guilliermondii* *C. kefyr* *C. krusei*

11	[42]	1994–1995	Belgium	Cross‐sectional	95/35	130	81	Oral	*C. albicans* *C. glabrata* *C. dubliniensis* *C. tropicalis* *C. krusei*

12	[45]	2020–2021	Iran	Cross‐sectional	168/108	276	113	Oral	*C. albicans* *C. glabrata* *C. dubliniensis* *C. tropicalis* *C. famata* *C. kefyr* *C. krusei* *C. parapsilosis* *C. africana* *C. stellatoidea*

13	[36]	1992–1994	France	Cohort	NA	1450	87	Esophageal	NA

14	[46]	1996–1997	Thailand	Cross‐sectional	230/48	278	110	Oral	NA

15	[39]	2020–2021	Nigeria	Cross‐sectional	106/194	300	40	Oral	*C. albicans* *C. krusei* *C. glabrata* *C. tropicalis*

Abbreviations: MC, mucosal candidiasis; NA, not available.

Among all included studies, 11 were cross‐sectional; two were cohort studies [35, 36]; and another two were case–control studies [37, 38]. These studies were conducted in North America [35], Africa [20, 39], South America [21, 40, 41], Europe [36, 42], and Asia [22, 37, 38, 43, 44, 45, 46].

Next, one examined esophageal candidiasis [36] and oropharyngeal candidiasis [22], while others investigated oral candidiasis.

Furthermore, seven studies [20–22, 39, 42, 44, 45] identified the *Candida* species as the etiological agent, and the remaining eight studies did not specify the *Candida* species.

The studies simultaneously reported both prevalence and potential risk factors of MC among PLWHA, as presented in Table 2. All studies contributed to the prevalence analysis, with reported prevalence ranging from 6.0% to 96.2%. The highest prevalence was reported by Tehrani et al. [38] (96.2%), followed by Schoofs et al. [42] (62.3%) and Menezes et al. [21] (60.5%), whereas the lowest prevalence was reported by Abgrall et al. [36] (6.0%), Schuman et al. [35] (11.9%), and Omosigho et al. [39] (13.3%). Commonly assessed variables included age, sex, CD4 count, antibiotic usage, smoking status, history of oral candidiasis, alcohol consumption, HIV clinical stage, viral load, highly active antiretroviral therapy (HAART) use, antibiotic use, denture use, smoking, alcohol consumption, duration since diagnosis, and hospitalization history.

**TABLE 2:** Prevalence and risk factors for MC across included studies.

No.	Study ID	Prevalence (%)	Risk factors																												
			AF	DP	VL	UD	PM	MT	SI	Sex	Age	Abx	Edu	Bru	CD4	Smo	His	Alc	Sev	Hos	BMI	Sta	Xer	Add	Loc	Occ	HAART	PrEg	Diag	Drug	Race
1	[20]	42.9								/	/	/	/	/	/												/	/	/		
2	[35]	11.9										/			/																
3	[21]	60.5	/	/	/					/	/	/			/	/	/										/				
4	[40]	50.0			/					/					/			/	/								/				
5	[43]	16.4								/					/												/		/		
6	[22]	51.4						/												/							/				
7	[38]	96.2								/	/		/			/		/			/						/		/	/	
8	[37]	46.2		/						/	/	/			/	/		/				/	/								
9	[41]	20.3								/					/																/
10	[44]	43.8				/	/	/	/	/	/													/							
11	[42]	62.3													/																
12	[45]	40.9				/		/	/	/	/		/		/						/	/		/						/	
13	[36]	6.0			/			/		/	/				/		/					/					/			/	
14	[46]	39.6	/								/										/										
15	[39]	13.3			/					/			/												/	/	/				

*Note:* DP: use of dental prostheses; MT: modes of HIV transmission; SI: shared injection practice; Abx: antibiotic usage; Edu: level of education; Bru: brush mouth once or twice daily; CD4: CD4 count; Smo: smoking status; His: history of oral candidiasis; Alc: alcohol consumption; Sev: severity of immunosuppression; Hos: hospitalization history; sta: HIV clinical stage; Xer: xerostomia; Add: addiction; Loc: location; Occ: occupation status; HAART: use of HAART; Preg: pregnancy; Diag: duration since diagnosis; Drug: intravenous drug use.

Abbreviations: AF, antifungal usage; PM, prophylactic medication; UD, underlying disease; VL, viral load.

### 3.3. Quality Assessment of the Included Studies

The quality assessment of the 15 included studies was assessed using the NOS for case–control, cohort, and cross‐sectional studies. The assessment of case–control studies showed fair quality (Table 3). For cohort studies, one was rated as good quality, while two were rated as fair quality (Table 4). For cross‐sectional studies, eleven studies were rated as fair quality, and three were rated as good quality (Table 5).

**TABLE 3:** Quality assessment for case–control studies.

No.	Study ID	Selection				Comparability	Outcome/exposure			Score (0–7)	Study^a^ quality
		S1	S2	S3	S4	C	O1	O2	O3		
1	[38]	^∗^			^∗^	^∗∗^	^∗^	^∗^		6	Fair
2	[37]	^∗^				^∗∗^	^∗^	^∗^		5	Fair

*Note:* S1: Is the case definition adequate? S2: representativeness of the cases. S3: selection of control. S4: definition of controls. C: comparability of cases and controls based on the design or analysis. O1: ascertainment of exposure. O2: same method of ascertainment for cases and controls. O3: nonresponse rate.

^a^A score of ≥ 7 is considered to have a “good quality” study, and a score of < 7 is considered to have a “fair quality” study.

^∗^The study met the high‐quality criterion for that item and received 1 star/score.

^∗∗^The study met both comparability criteria and received 2 stars/scores.

**TABLE 4:** Quality assessment for cohort studies.

No.	Study ID	Selection				Comparability	Outcome/exposure			Score (0–7)	Study^a^ quality
		S1	S2	S3	S4	C	O1	O2	O3		
1	[35]	^∗^	^∗^			^∗∗^	^∗^	^∗^	^∗^	7	Good
2	[21]	^∗^	^∗^			^∗^	^∗∗^	^∗^		6	Fair
3	[36]	^∗^	^∗^			^∗^	^∗^			4	Fair

*Note:* S1: representativeness of the exposed cohort. S2: selection of the nonexposed cohort. S3: ascertainment of exposure. S4: demonstration that the outcome of interest was not present at the start of the study. C: comparability of cohorts based on the design or analysis. O1: assessment of outcome. O2: Was follow‐up long enough for outcomes to occur? O3: adequacy of follow‐up of cohorts.

^a^A score of ≥ 7 is considered to have a “good quality” study, and a score of < 7 is considered to have a “fair quality” study.

^∗^The study met the high‐quality criterion for that item and received 1 star/score.

^∗∗^The study met both comparability criteria and received 2 stars/scores.

**TABLE 5:** Quality assessment for cross‐sectional studies.

No.	Study ID	Selection				Comparability	Outcome		Score (0–7)	Study^a^ quality
		S1	S2	S3	S4	C	O1	O2		
1	[20]	^∗^	^∗^			^∗∗^	^∗∗^	^∗∗∗^	7	Good
3	[22]	^∗^	^∗^			^∗^	^∗∗^	^∗^	6	Fair
4	[42]	^∗^	^∗^			^∗^	^∗^		4	Fair
6	[45]	^∗^	^∗^			^∗∗^	^∗∗^	^∗^	7	Good
7	[39]	^∗^	^∗^			^∗^	^∗^	^∗^	5	Fair
8	[43]	^∗^	^∗^				^∗∗^	^∗^	5	Fair
9	[40]	^∗^	^∗^			^∗∗^	^∗^	^∗^	6	Fair
10	[44]	^∗^			^∗^	^∗∗^	^∗^	^∗^	7	Good
12	[41]	^∗^			^∗^	^∗^	^∗^	^∗^	5	Fair
13	[46]	^∗^			^∗^	^∗^	^∗∗^	^∗^	6	Fair

*Note:* S1: truly representative (all subjects or random sampling) or somewhat representative (nonrandom sampling) of the average in the target population. S2: sample size (justified and satisfactory). S3: comparability between respondent’s and nonrespondent’s characteristics is established, and the response rate is satisfactory. S4: ascertainment of the exposure (risk factor) using a validated measurement tool. C: The subjects in different outcome groups are comparable, based on the study design or analysis. Confounding factors are controlled. O1: assessment of the outcome using independent blind assessment or record linkage. O2: The statistical test used to analyze the data is clearly described and appropriate, and the measurement of the association is presented, including confidence intervals and the probability level (*p*‐value).

^a^A score of ≥ 7 is considered to have a “good quality” study, and a score of < 7 is considered to have a “fair quality” study.

^∗^The study met the high‐quality criterion for that item and received 1 star/score.

^∗∗^The study met both comparability criteria and received 2 stars/scores.

### 3.4. Overall Pooled Prevalence of MC in PLWHA

**FIGURE 2:**
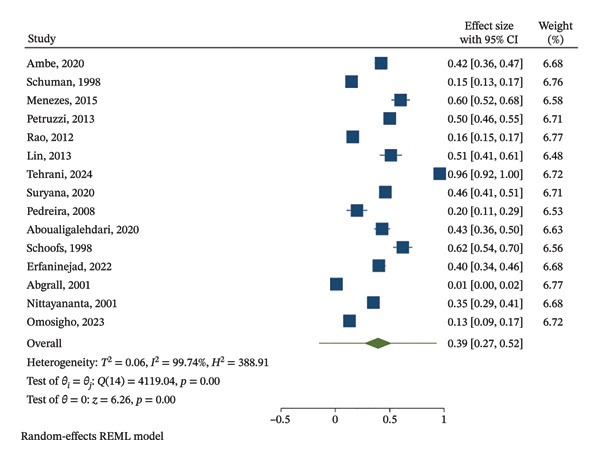
Overall pooled prevalence of MC in PLWHA.

A total of 15 studies provided the prevalence of MC in PLWHA. The global overall pooled prevalence was 39.0% (95% CI: 27%–52%; *I* ^2^  = 99.74%, *p* = 0.001) (Figure 2).

### 3.5. Overall Pooled Prevalence of MC Among PLWHA in High‐Quality Articles

**FIGURE 3:**
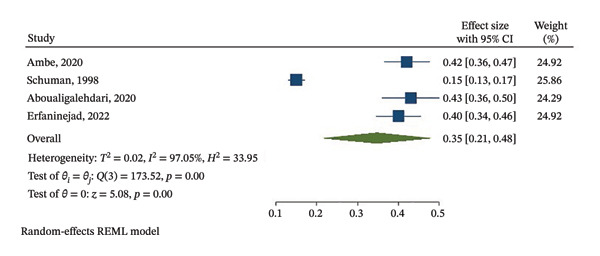
Overall pooled prevalence of MC among PLWHA in high‐quality articles.

Of the 15 studies, four were rated as high‐quality articles based on NOS. The pooled prevalence of MC in these studies was 35.0% (95% CI: 21%–48%; *I* ^2^ = 97.05%, *p* = 0.001) (Figure 3).

### 3.6. Overall Pooled Prevalence of MC in PLWHA by Geographical Region

Of the 15 included articles, seven were conducted in Asia, three in South America, two in Africa and Europe, and one in North America. As only one study was available from North America, the overall pooled prevalence analysis was not performed for this region.

**FIGURE 4:**
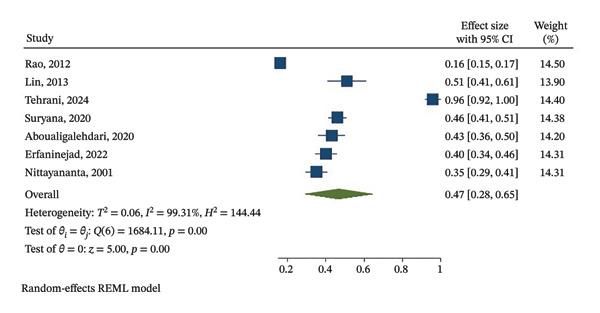
Overall pooled prevalence of MC among PLWHA in Asia.

**FIGURE 5:**
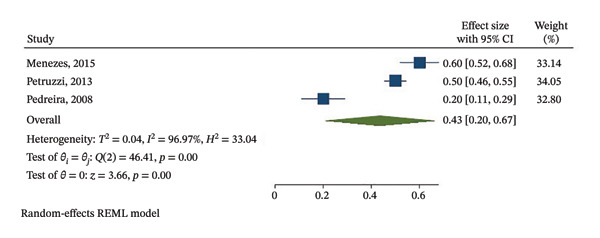
Overall pooled prevalence of MC in PLWHA in South America.

The overall pooled prevalence of MC among PLWHA in Asia was 47.0% (95% CI: 28%–65%; *I* ^2^ = 99.31%, *p* = 0.001), 43.0% in South America (95% CI: 20%–67%; *I* ^2^ = 96.97%, *p* = 0.001), 31.0% in Europe (95% CI: −28%–91%; *I* ^2^ = 99.49%, *p* = 0.001), and 27.0% in Africa (95% CI: −1%–56%; *I* ^2^ = 96.57%, *p* < 0.001) (Figures 4 and 5). The negative lower bound was observed for Africa and the European region, likely due to the huge prevalence differences between the two studies, with values ranging from 1% to 65% in Europe and 13% to 50% in Africa. Therefore, both regions were excluded from this analysis.

### 3.7. Overall Pooled Prevalence of Oral Candidiasis in PLWHA

**FIGURE 6:**
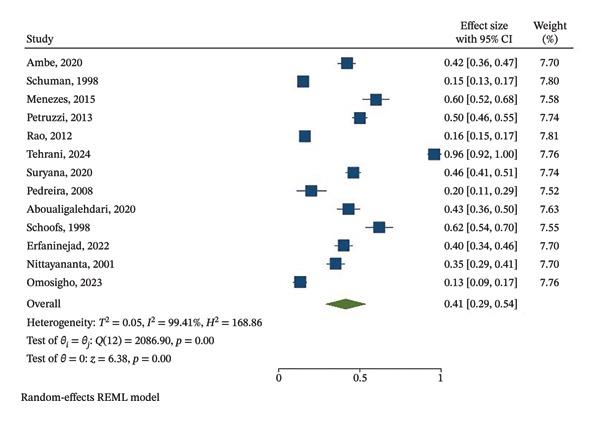
Overall pooled prevalence of oral candidiasis in PLWHA.

Among the included studies, 13 studied oral candidiasis in PLWHA, one studied esophageal candidiasis, and one studied oropharyngeal candidiasis. Therefore, an overall prevalence was performed for the oral candidiasis, yielding 41.0% in PLWHA (95% CI: 29%–54%; *I* ^2^ = 99.41%, *p* = 0.001) (Figure 6).

### 3.8. Assessment of Publication Bias

Publication bias was assessed only when the analysis contained more than 10 articles [34]. Consequently, geographical region subgroup analysis was excluded. In this study, the publication bias was assessed for the overall pooled prevalence of (i) MC in PLWHA and (ii) oral candidiasis in PLWHA. The Egger test disclosed no publication bias for the overall pooled prevalence of MC (*p* = 0.072) and oral candidiasis in PLWHA (*p* = 0.318).

**FIGURE 7:**
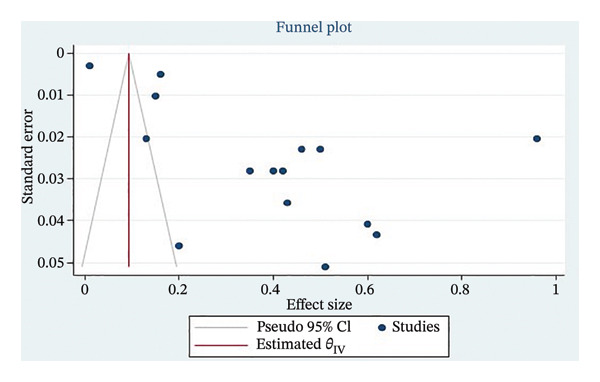
Funnel plot test for the overall pooled prevalence of MC in PLWHA.

**FIGURE 8:**
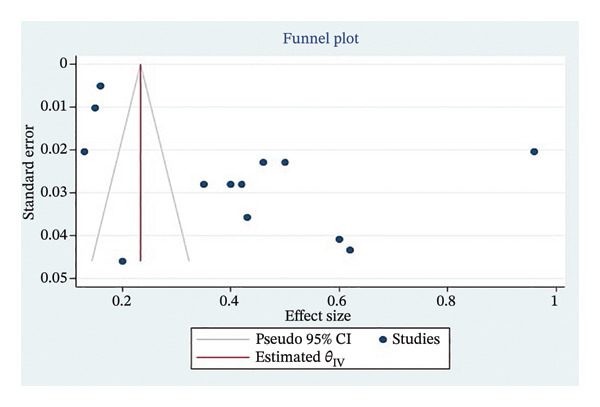
Funnel plot test for the overall pooled prevalence of oral candidiasis in PLWHA.

Funnel plot assessment for both analyses (Figures 7 and 8) suggested possible visual asymmetry, indicating a potential risk of publication bias. However, Egger’s regression test was not statistically significant (*p* > 0.05), and the intercept values were close to zero, suggesting no strong evidence of asymmetry. Therefore, there is no statistically significant evidence of publication bias.

The discrepancy between visual inspection and statistical testing may be attributed to the small number of included studies (*n* = 15), which reduces the power of Egger’s test. In addition, funnel plot asymmetry may arise from clinical or methodological heterogeneity, small‐study effects, or random variation rather than true publication bias. Notably, the *p*‐value for Figure 7 (*p* = 0.072) may be considered borderline; however, it does not reach statistical significance and should be interpreted cautiously.

### 3.9. Sensitivity Analysis

The sensitivity analysis was performed using the leave‐one‐out method for the overall prevalence of MC in PLWHA to evaluate the influence of individual studies on the pooled estimate and heterogeneity (Table 6). The results indicated that omission of any study did not substantially change the pooled prevalence estimate, although heterogeneity remained high across the analyses. When Pedreira et al. [41] were excluded, the adjusted pooled prevalence was 0.41 (95% CI: 0.28–0.53), with high heterogeneity (*I* ^2^ = 99.77). The Egger test *p*‐value in this leave‐one‐out analysis was 0.0149, suggesting potential publication bias. A similar finding was observed when the study by Tehrani et al. [38] was excluded (*p* = 0.0005). Overall, the pooled prevalence estimates remained relatively stable across the leave‐one‐out analyses, while Egger’s test results varied after the exclusion of specific studies.

**TABLE 6:** Sensitivity analysis for the overall prevalence with the leave‐one‐out method.

No	If excluded	Effect size with 95% CI	*I* ^2^	Egger’s test
1	Ambe et al. [20]	0.39 (0.26, 0.52)	99.77	0.0847
2	Schuman et al. [35]	0.41 (0.28, 0.54)	99.72	0.1404
3	Menezes et al. [21]	0.38 (0.25, 0.51)	99.76	0.1201
4	Petruzzi et al. [36]	0.38 (0.25, 0.52)	99.77	0.0727
5	Rao et al. [37]	0.41 (0.28, 0.54)	99.49	0.1532
6	Lin et al. [22]	0.38 (0.25, 0.51)	99.77	0.0919
7	Tehrani et al. [38]	0.35 (0.25, 0.45)	99.60	0.0005
8	Suryana et al. [39]	0.39 (0.26, 0.52)	99.77	0.0772
9	Pedreira et al. [40]	0.41 (0.28, 0.53)	99.77	0.0149
10	Aboualigalehdari et al. [41]	0.39 (0.26, 0.52)	99.78	0.0847
11	Schoofs et al. [42]	0.38 (0.25, 0.50)	99.76	0.1326
12	Erfaninejad et al. [43]	0.39 (0.26, 0.52)	99.77	0.0845
13	Abgrall et al. [44]	0.42 (0.30, 0.54)	99.33	0.2668
14	Nittayananta et al. [45]	0.40 (0.26, 0.53)	99.77	0.0829
15	Omosigho et al. [46]	0.41 (0.29, 0.54)	99.75	0.0972

*Note:* The “Effect Size with 95% CI” represents the adjusted pooled prevalence and its 95% confidence interval after excluding each individual study one at a time. *I* ^2^ indicates the level of heterogeneity, and the Egger test *p*‐value assesses potential publication bias after each study removal.

### 3.10. Meta‐Regression

**FIGURE 9:**
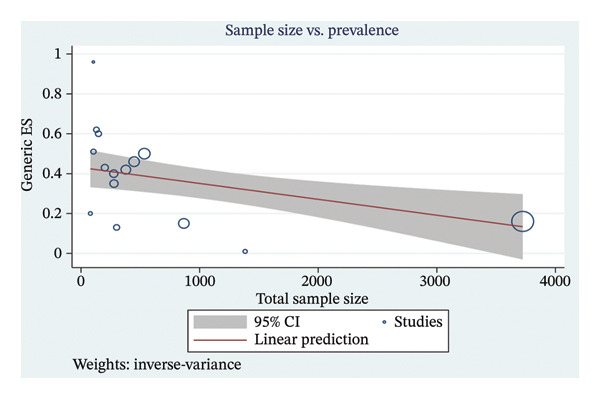
Bubble plot analysis for the overall prevalence results.

As high heterogeneity (*I* ^2^ > 75%) was found in the overall pooled prevalence of MC in PLWHA, a meta‐regression was performed to explore the potential sources of heterogeneity across studies. Sample size was identified as a statistically significant moderator of the overall pooled prevalence (Wald *χ* ^2^ = 8.06, *p* = 0.005). A negative association was observed between sample size and logit prevalence, indicating that smaller studies tended to report higher prevalence estimates, whereas larger studies reported lower estimates. Sample size explained 66.8% of the between‐study heterogeneity, with residual heterogeneity decreasing to *I* ^2^ = 22.3% (*τ* ^2^ = 0.0052; Q_res = 16.44, Prob > Q_res = 0.226). This pattern was also supported by the bubble plot, which showed a downward trend (Figure 9).

Geographic region was also assessed as a moderator for the overall pooled prevalence of MC; however, it was not statistically significant based on the Wald test (Wald *χ* ^2^ = 2.18, *p* = 0.536). Residual heterogeneity remained moderate after meta‐regression (*τ* ^2^ = 0.0155; *I* ^2^ = 50.83%; Q_res = 26.09, Prob > Q_res = 0.006), suggesting that geographic region did not fully explain the between‐study variability.

For oral candidiasis prevalence, meta‐regression showed that geographic region was a statistically significant moderator (Wald *χ* ^2^ = 33.1, *p* < 0.001). Residual heterogeneity was no longer detected after meta‐regression (*τ* ^2^ = 1.8e−07; *I* ^2^ = 0%; Q_res = 0.00, Prob > Q_res = 1.000), suggesting that geographic region explained the between‐study variability in this analysis. The detailed meta‐regression results, including the meta‐regression coefficient, SE, value, *p*‐value, and 95% CI, are presented in Table 7.

**TABLE 7:** Meta‐regression analysis of potential sources of heterogeneity in the pooled prevalence of MC and oral candidiasis among PLWHA.

Outcome	Moderator	*k*	Wald *χ* ^2^	Prob > chi^2^	Meta‐regression coefficient	SE	*z*‐value	95% CI	*I* ^2^
Overall MC prevalence	Sample size	15	8.06	0.005	−0.0000798	0.0000281	−2.84	−0.0001349 to −0.0000247	22.3
Overall MC prevalence	Geographic region	15	2.18	0.536	0.4099	0.0851	4.81	0.2431 to 0.5768	50.8
Oral candidiasis prevalence	Geographic region	13	33.1	< 0.001	1.0000	0.1737	5.75	0.6593841 to 1.340616	0

*Note:* *τ* ^2^: between‐study variance; Q_res: residual heterogeneity statistic. The Wald *χ* ^2^ test indicates the overall significance of each moderator. Meta‐regression coefficients represent associations with logit‐transformed prevalence.

Abbreviations: CI, confidence interval; MC, mucocutaneous candidiasis; PLWHA, people living with HIV/AIDS; SE, standard error.

### 3.11. Risk Factors for MC in PLWHA

Sixteen risk factors of MC in PLWHA are summarized in Table 3.

#### 3.11.1. Gender

**FIGURE 10:**
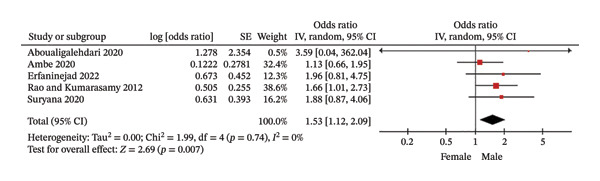
Meta‐analysis of gender as a risk for MC in PLWHA.

Of 15 studies, seven studies investigated gender as a risk factor for MC in PLWHA. Five studies [20, 37, 43, 44, 45], which investigated oral candidiasis in PLWHA, have provided sufficient data and were included in this meta‐analysis (Figure 10). Based on the result, males have a significantly higher risk of developing oral candidiasis compared to females (pooled OR 1.53, 95% CI: 1.12–2.09, *p* = 0.007).

Two additional studies, Tehrani et al. [38] and Petruzzi et al. [40], could not be included in this pooled estimate as they used an inverted comparison (female versus male), finding a nonsignificant effect OR.

#### 3.11.2. Age

Seven studies examined age as a potential risk factor for oral candidiasis with one additional study examining esophageal candidiasis, with highly heterogeneous findings. Six studies, namely Ambe et al. [20], Menezes et al. [21], Tehrani et al. [38], Aboualigalehdari et al. [44], Erfaninejad et al. [45], and Nittayananta et al. [46], found no significance between oral candidiasis and PLWHA. In comparison, Suryana et al. [37] found that younger age (< 34 vs. ≥ 34 years) was significantly associated with oral candidiasis (OR = 0.66, *p* = 0.03). Abgrall et al. [36] linked the 17–69 age group to esophageal candidiasis development (*p* = 0.02). Overall, the evidence does not support a consistent or strong association between age and MC risk in this population.

#### 3.11.3. Education Level

Four studies linked the association between lower education level and higher odds of oral candidiasis. Ambe et al. [20] found a nonsignificant OR for primary (OR = 1.22, *p* = 0.628) and secondary (OR = 1.44, *p* = 0.380) education compared to university‐level education. In contrast, Tehrani et al. [38] found a significant association for secondary and higher education versus elementary education (OR = 0.032, *p* < 0.001). Erfaninejad et al. [45] observed nonsignificant associations (*p* = 0.071) between primary/middle school (OR = 0.666) and secondary/higher education (OR = 0.394) levels against illiteracy with PLWHA. Omosigho et al. [39] identified a significant association across primary, secondary, and tertiary education levels (*p* < 0.001).

#### 3.11.4. Local Oral Environment

Four studies examined factors related to the local oral environment. Oral hygiene behavior was identified as a significant risk factor in two studies. Ambe et al. [20] found brushing once compared to twice daily increased odds of infection (OR = 1.92, 95% CI: 1.25–2.94, *p* = 0.002), and Suryana et al. [37] observed xerostomia as a significant risk factor (OR = 4.15, 95% CI: 2.76–6.23, *p* < 0.05). In comparison, the evidence for dental prostheses was mixed. Menezes et al. [21] noted a significant positive association (*p* = 0.017); however, Suryana et al. [37] found no significant effect (OR = 1.25, 95% CI: 0.81–1.93, *p* = 0.311).

#### 3.11.5. *Candida* Species

Among seven studies that have identified *Candida* species [20–22, 39, 42, 44, 45], *C. albicans* was the most frequently isolated species in each of the studies. However, the prevalence of non–*C. albicans* species is also substantial. Specifically, *C. tropicalis* and *C. glabrata* were also observed in every included study (*n* = 7), while *C. krusei* identified in six studies. Other species, including *C. dubliniensis*, *Candida parapsilosis*, and other rare species, were identified in four to five studies, reflecting a varied and less frequent distribution compared to other dominant species.

#### 3.11.6. Geographical Regions

The majority of included studies originated from Asia (India [43], Taiwan [22], Iran [38, 44, 45], Indonesia [37], and Thailand [46]), followed by South America (Brazil [21, 40, 41], Africa [Cameroon [20], Nigeria [39]}, Europe [Belgium [42], France [36]], and North America [United States [35]]). This distribution reflects the global burden of HIV‐associated MC, showing a research concentration in Asian and South American populations. All geographical regions primarily investigated oral candidiasis, while Europe contributed studies specifically on oropharyngeal [42] and esophageal candidiasis [36].

#### 3.11.7. Antibiotic Usage

**FIGURE 11:**
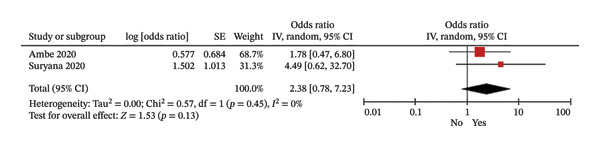
Meta‐analysis of antibiotic use as a risk for MC in PLWHA.

Four oral candidiasis studies examined the impact of antibiotic usage in PLWHA. A meta‐analysis by Ambe et al. [20] and Suryana et al. [37] yielded a pooled OR of 2.38 (95% CI: 0.78–7.23, *p* = 0.130), indicating that antibiotic use was not significantly associated with MC in PLWHA (Figure 11).

However, two additional studies could not be included in the meta‐analysis due to incompatible data reporting. Menezes et al. [21] identified a significant association (OR = 3.2, *p* = 0.025) but did not provide sufficient data, including CI. Similarly, Schuman et al. [35] only described antibiotic use as a significant risk factor (*p* < 0.001). Notably, all studies consistently identified antibiotic usage as a risk factor.

#### 3.11.8. CD4 Count

**FIGURE 12:**
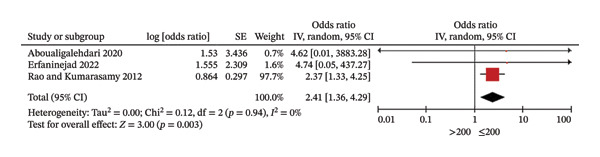
Meta‐analysis of CD4 count (≤ 200 versus > 200 cells/µL) as a risk for MC in PLWHA.

CD4 count was investigated in 10 studies involving oral candidiasis, but only three studies [43, 44, 45] used the identical comparison reference (≤ 200 cells/µL versus > 200 cells/µL) for oral candidiasis. The meta‐analysis found that a CD4 count below 200 cells/µL was a significant risk factor for oral candidiasis (pooled OR 2.41, 95% CI: 1.36–4.29, *p* = 0.003) (Figure 12).

The remaining seven studies, which could not be included in the pooled estimate due to differing thresholds or reporting formats, uniformly supported this association. Three studies provided significant ORs using alternative CD4 thresholds. For example, Suryana et al. [37] described < 108 versus ≥ 108 cells/µL (OR = 3.29); Tehrani et al. [38] identified > 200 versus < 200 cells/µL (OR = 0.12); and Petruzzi et al. [40] reported < 350 versus > 500 cells/µL (OR = 4.88). The final four studies [21, 35, 41, 42], which provided only percentages or *p*‐values, also consistently identified lower CD4 counts as a significant risk factor. Despite methodological heterogeneity in measurement, all 10 studies consistently demonstrate that poorer immunologic status, indicated by lower CD4 counts, significantly increases the risk of MC.

#### 3.11.9. Viral Load

One study examined the association between viral load and esophageal candidiasis in PLWHA, while four additional studies investigated oral candidiasis. Abgrall et al. [36] noted a significant association of 50,000 copies/mL viral load on esophageal candidiasis. For oral candidiasis, Petruzzi et al. [40] identified significant associations across different categories of viral loads: 51–5000 copies/mL, 5001–20,000 copies/mL, and > 20,000 copies/mL (ORs = 2.54–3.72, all *p* < 0.001). Additionally, Omosigho et al. [39] observed a significant association of viral load, wherein 65% of their study included PLWHA with 10–1000 copies/mL (*p* < 0.001). Tehrani et al. [38] found higher viral load (7.33 × 10^5^ ± 1.31 × 10^6^) in PLWHA with oral candidiasis carried OR = 8.00 (*p* < 0.001). However, Menezes et al. [21] found no significance when comparing colonized and noncolonized *Candida* spp. in the oral cavity (*p* = 0.30). Generally, higher viral load was consistently associated with increased risk.

#### 3.11.10. HIV Clinical Stage

Four studies identified advanced disease stage as a risk factor in oral and oropharyngeal candidiasis in PLWHA. Suryana et al. [37] noted a significant association for stage 2–4 versus stage 1 (OR = 3.58, *p* < 0.05). In contrast, Erfaninejad et al. [45] found no significant association for stages 2, 3, and 4 versus stage 1 (*p* = 0.05). Nittayananta et al. [46] noted significance for symptomatic stages (*p* < 0.05), and Abgrall et al. [36] observed significance between AIDS and asymptomatic stages in oropharyngeal candidiasis (*p* < 0.05).

#### 3.11.11. Duration Since HIV Diagnosis

Three studies investigated the association of duration since HIV diagnosis with oral candidiasis in PLWHA. Rao et al. [43] identified that a duration of more than 6 years (versus ≤ 6 years) was associated with higher odds of candidiasis (OR = 1.36, 95% CI: 1.07–1.72, *p* = 0.012). In contrast, Ambe et al. [20] found no significant association for a diagnosis made less than 5 years versus ≥ 5 years (OR = 0.90, 95% CI: 0.60–1.35), and Tehrani et al. [38] also documented a nonsignificant effect (OR = 0.92, 95% CI: 0.85–1.00, *p* = 0.158). The evidence is therefore inconsistent, with one study showing a significant positive association and two demonstrating no significant effect.

#### 3.11.12. HAART

**FIGURE 13:**
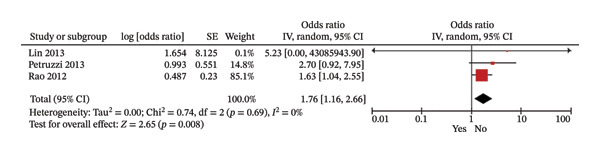
Meta‐analysis of HAART as a risk for MC in PLWHA.

Eight studies examined the association between antiretroviral therapy and oral, oropharyngeal, and esophageal candidiasis in PLWHA. The meta‐analysis of oropharyngeal candidiasis [22] and oral candidiasis [40, 43] revealed that PLWHA not receiving HAART were more likely to develop MC compared to the treated person (pooled OR 1.76; 95% CI: 1.16–2.66; *p* = 0.008) (Figure 13).

The remaining five studies could not be included in this meta‐analysis due to different analytical approaches. Tehrani et al. [38] noted a significant OR with 0.005. However, one oral study [21] found nonsignificant associations with OR = 1.51. Two studies on oral candidiasis [20, 39] and one on esophageal candidiasis [20, 36, 39] included HAART but reported only significant *p*‐values. Collectively, it consistently indicates that inadequate HAART will increase MC risk in PLWHA.

#### 3.11.13. Intravenous Drug Use

**FIGURE 14:**
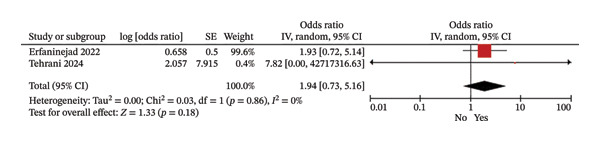
Meta‐analysis of drug abuse as a risk factor for MC in PLWHA.

Three studies investigated this risk factor on oral candidiasis in PLWHA. A meta‐analysis of two studies [38, 45] that provided sufficient quantitative data yielded a pooled OR of 1.94 (95% CI: 0.73–5.16, *p* = 0.18), with no statistically significant association observed (Figure 14).

The third study, Aboualigalehdari et al. [44], which could not be included in the meta‐analysis due to differences in reporting, found no significant association (OR = 0.78, *p* = 0.697). The evidence from the available studies suggests that intravenous drug use is not significantly associated with MC in PLWHA.

#### 3.11.14. Alcohol Consumption

**FIGURE 15:**
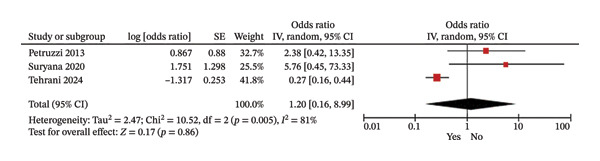
Meta‐analysis of alcohol consumption as a risk factor for MC in PLWHA.

Three studies examined alcohol consumption on oral candidiasis in PLWHA [37, 38, 40]. The meta‐analysis demonstrated that alcohol consumption was not a significant risk factor for the development of oral candidiasis (pooled OR 1.20; 95% CI: 0.16–8.99; *p* = 0.86) (Figure 15).

#### 3.11.15. Sharing Injection

**FIGURE 16:**
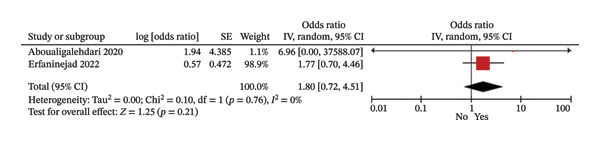
Meta‐analysis of shared injection as a risk factor for MC in PLWHA.

Two studies [44, 45] assessed the risk factor of sharing injection devices on oral candidiasis. Meta‐analysis showed no significant association between sharing an injection device and oral candidiasis development compared to a person who did not share (pooled OR 1.80; 95% CI: 0.72–4.51; *p* = 0.21) (Figure 16).

#### 3.11.16. Smoking

Three studies documented conflicting results on the effect of smoking on oral candidiasis in PLWHA. Suryana et al. [37] noted a significant association (OR = 6.83, *p* < 0.05). However, Menezes et al. [21] and Tehrani et al. [38] found no significant association (*p* > 0.05).

## 4. Discussion

MC in PLWHA has been a global concern for decades. However, few studies documented the pool or overall prevalence of MC, particularly oral candidiasis among PLWHA across the regions. In this review, we pooled all the prevalence of oral candidiasis across the regions. Our findings presented a 39% overall pooled prevalence of MC in PLWHA, with oral candidiasis at 41%. Asia had the highest MC prevalence (47%) compared with the lowest in Africa (27%). This finding is comparable to findings reported by previous studies, including Erfaninejad et al. [45] and Nittayananta et al. [46]. However, it is higher than several studies, such as Schuman et al. [35] and Abgrall et al. [36], while lower than others, including Menezes et al. [21] and Tehrani et al. [38]. These differences may be attributed to variations in patient characteristics, immune status, and healthcare access across study settings. Next, meta‐regression analysis identified that sample size significantly influenced prevalence estimates, with larger studies reporting lower logit prevalence. The result shows the existence of small‐study effects, whereby smaller studies may overestimate prevalence. However, the sample size is not crucial for epidemiological determinants; therefore, this association may reflect methodological or reporting biases rather than a true population‐level effect.

Demographic factors revealed distinct risk patterns. Our meta‐analysis indicated that male gender is significantly associated with oral candidiasis in PLWHA. Possible explanations include the higher prevalence of HIV in males [47, 48] as well as gender differences in hazardous behaviors, such as tobacco or alcohol use, compared to women, potentially leading to increased susceptibility [42, 49–51]. However, age exhibited an inconsistent association across studies, indicating that other factors might predominate over age‐related susceptibility. Lower educational attainment is correlated with the elevated risk, plausibly serving as a surrogate for socioeconomic mediators, including health literacy, healthcare access, and therapeutic adherence, rather than exerting direct biological effects. These findings highlight that education could be a key modifiable contributor alongside gender.

Next, the local oral environment can directly influence physiological processes, offering actionable targets for clinical intervention. Our narrative synthesis identified both poor oral hygiene and xerostomia as significant risk factors in individual studies. This underscores that local mucosal integrity and microbial flora are the frontline defense against candidiasis [52, 53]. The clinical implication is direct where integrating the routine oral assessment and hygiene promotion into standard HIV care could mitigate a modifiable risk pathway. However, the current evidence is derived from a small number of studies; it highlights a need for more robust investigation on specific oral health practices and their direct impact on candidiasis incidence in this population.

Antibiotic use represents a well‐established risk factor for candidiasis in PLWHA through mechanisms, including disruption of protective microbial flora. Previous studies highlighted that antibiotic treatment can decrease the thickness of the colonic mucus layer [54], suppress bacterial flora, and disrupt its normal balance [55, 56]. The prolonged use of broad‐spectrum antibiotics thereby increased the colonization and proliferation of *Candida* spp. and heightened the risk of developing MC [57]. Similarly, our narrative synthesis consistently identified antibiotic use as a risk factor for oral candidiasis in PLWHA. We therefore emphasize that while antibiotic stewardship remains important in PLWHA care, its specific role as an independent risk factor for MC in this population requires further clarification through standardized prospective studies.

Despite the paucity of data for quantitative pooling, the narrative synthesis reveals highly consistent findings. For example, both low CD4 count and lack of HAART were strongly associated with increased MC risk. The low CD4 counts with impaired T cell–mediated defense against *Candida* species, elevating oral and esophageal infection rates in PLWHA [58]. Similarly, our finding underscores the importance of antiretroviral therapy in restoring immune competence. These factors are intrinsically linked, as HAART primarily exerts its protective effect by improving CD4 counts and reducing viral load [59, 60]. Our findings reinforce current clinical guidelines advocating for early HAART initiation regardless of CD4 count, as this approach addresses the fundamental immune impairment underlying candidiasis risk.

While elevated viral load is consistently associated with candidiasis risk, this relationship is likely mediated through its causal role in CD4+ T‐cell depletion [61]. Similarly, the association of advanced WHO clinical stage may also reflect its correlation with the current degree of immunosuppression rather than an independent contribution of the clinical events. In contrast, duration of HIV infection might not be a reliable risk factor, suggesting that an individual’s current immunologic status is a more direct and reliable determinant of candidiasis risk than the historical length of infection. These findings highlight that CD4 count could represent the primary pathogenic pathway, with viral load and clinical stage as correlated rather than independent predictors.

Among behavioral factors, intravenous drug use and sharing injection device demonstrated no significant associations with MC in the majority of relevant studies. Alcohol consumption also showed positive association, though with highly variable effect sizes. However, these findings are based on only two or three studies per factor, and exposure definitions were inconsistent. The limited evidence, along with overlapping socioeconomic and clinical factors, prevents solid conclusions about whether these are independent risk factors. Future studies with standardized behavioral assessments and comprehensive adjustment are warranted.

Other factors with plausible biological links, including smoking, diabetes mellitus, and steroid use, did not demonstrate clear, consistent relationships with candidiasis in PLHWA across studies. These inconsistencies highlight important research gaps, including standardized definitions of exposures, prospective assessment in diverse HIV populations, and evaluation of whether these factors modify the effects of core immune parameters, such as CD4 count. In clinical practice, routine antifungal prophylaxis is not recommended for PLWHA even if they have a CD4 count below 200 cells/uL, because early initiation of HAART can reverse or prevent the development of MC [24]. However, the role of antifungal prophylaxis could not be addressed in our review due to insufficient data in the included PLWHA studies, representing another important area for future research.

In addition, the geographical distribution of included studies reveals significant regional representation disparities, with 47% of evidence originating from Asian populations and only 27% from Africa. This uneven distribution may introduce geographic bias and affect the generalizability of risk factor estimates across different healthcare systems and genetic populations. Moreover, the concentration of studies in specific regions, such as Iran and Brazil, each with three studies, suggests possible clustered research efforts rather than uniform global surveillance.

Furthermore, regional variations in *Candida* species distribution were observed. Asian studies report greater diversity of non–*C. albicans* species compared to other regions, which may potentially change the treatment strategy. Three included studies have provided the antifungal susceptibility of their isolated *Candida* spp. [20, 22, 42]. Two showed high fluconazole susceptibility in *C. albicans* (MIC_90_ = 1 µg/mL), contrasted by significant resistance in non–*C. albicans* species, especially *C. tropicalis* and *C. glabrata*, with MIC_90_ up to 64 µg/mL. These geographic patterns highlight the need for more balanced multinational studies to represent global HIV‐positive populations and address potential region‐specific risk factors and pathogen distributions.

In this study, the grouping of distinct anatomical sites as MC acknowledges their shared etiology while recognizing potential pathophysiological differences. Given the predominance of oral candidiasis studies (13/15), our findings primarily apply to this site. The single esophageal candidiasis study observed more profound CD4 depletion, suggesting a severity gradient. However, core immune factors (CD4 and HAART) remained relevant across sites, even though site‐specific studies are required to clarify the unique risk profile.

**FIGURE 17:**
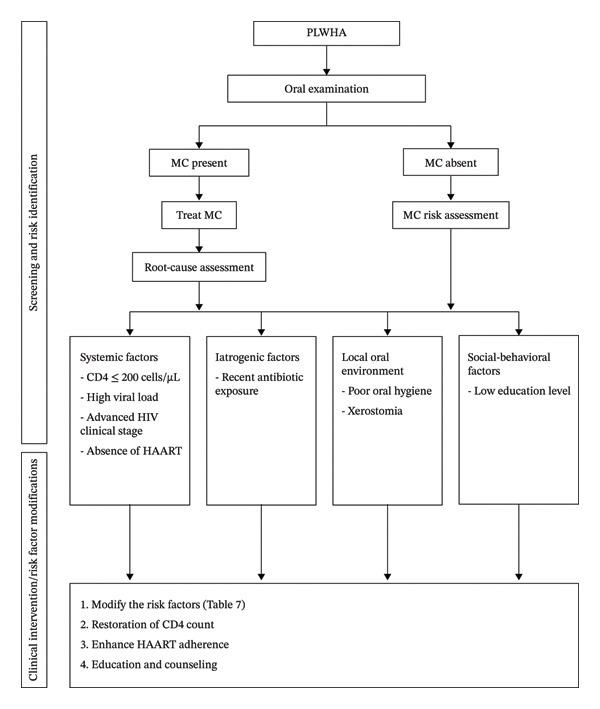
Risk factors and clinical intervention for MC in PLWHA.

Following this, we propose a screening algorithm (Figure 17) to identify PLWHA at high risk of MC and risk modification strategies (Table 8) emphasizing CD4 restoration and HAART adherence. However, future cost‐effectiveness analysis is needed to compare the absolute risk reduction of antifungal prophylaxis versus standard care in PLWHA. Our findings, which affirm that immunosuppression and inadequate HAART are the primary modifiable risk factors, therefore reinforce the existing guideline‐recommended strategy of optimizing antiretroviral therapy, which could be the fundamental cost‐effective preventive measure.

**TABLE 8:** Clinical recommendations for MC in PLWHA by identified risk factors.

Risk factor category	Clinical recommendation
Systemic causes (low CD4, high viral load, HAART treatment initiation, and HIV clinical stage)	Prioritize achieving viral suppression with effective HAART. Perform routine oral examination at every encounter in patients with low CD4 counts (≤ 200 cells/µL).
Iatrogenic factor (recent antibiotic exposure)	Justify the clinical indication for all antibiotic prescriptions; this strategy aims to reduce the incidence and recurrence of oral candidiasis.
Local oral environment (poor hygiene or xerostomia)	Reinforce oral hygiene self‐care with repeated patient education.
Social–behavioral factors (low education level)	Screen for substance abuse and refer for counseling, rehabilitation, or addiction program.
Gender (male)	Although males are a significant factor in MC development among PLWHA, this is likely due to their larger role in the HIV epidemic. As a result, screening should not be biased by gender. All patients, both male and female, with the aforementioned risk factors should be reviewed.

Several limitations constrain the interpretation of this review. Despite focusing primarily on oral candidiasis which limits the generalizability and applicability to oropharyngeal or esophageal candidiasis in PLWHA, additional constraints include heterogeneity in HIV clinical stage and CD4 count thresholds across studies of 79–3724 participants, which may disproportionately influence the pooled estimates. In addition, the geographic distribution of included studies is also uneven, with a majority originating from Asia (7/15), hence limiting the generalizability of findings to other global regions with different HIV epidemiology and care standards. These factors collectively highlight the need for more standardized, prospective, and globally representative research.

## 5. Conclusion

In conclusion, this review revealed the global overall pooled prevalence of MC of 39.0% with Asia showing the highest estimate in PLWHA. Next, male gender, CD4 counts below 200 cells/µL, and the absence of HAART are significantly associated with MC in PLWHA. However, the impact of other potential risk factors, including poor oral hygiene, lower education level, higher viral load, and advanced HIV clinical stage, requires further investigation.

## Funding

This study was supported by the Ministry of Health Malaysia Research grant (NMRR ID 23‐01040‐JHL (IIR)).

## Conflicts of Interest

The authors declare no conflicts of interest.

## Supporting Information

Additional supporting information can be found online in the Supporting Information section.

## Supporting information


**Supporting Information** Supporting Table 1. Search strategy and results by Medline database. Supporting Table 2. Search strategy and results by Embase database. Supporting Table 3. Search strategy and results by Central database. Supporting Table 4. Search strategy and results by Scopus database.

## Data Availability

All data were provided with references in the text and the reference list.
