# Supplementary material for: Prevalence and Risk Factors for Oropharyngeal, Esophageal, and Oral Candidiasis in HIV‐Positive Individuals: A Systematic Review and Meta‐Analysis
Source: Interdiscip Perspect Infect Dis. 2026 Jul 29;2026:6445594. doi: 10.1155/ipid/6445594 (PMC13417023; doi:10.1155/ipid/6445594)
Supplement: Supplementary file 1 — Supporting Information Supporting Table 1. Search strategy and results by Medline database. Supporting Table 2. Search strategy and results by Embase database. Supporting Table 3. Search strategy and results by Central database. Supporting Table 4. Search strategy and results by Scopus database. [file IPID-2026-6445594-s001.docx]

**Appendix A**

Supplementary TABLE 1. Search strategy and results by Medline database

| **No.** | **Search Terms** | **Results** |
| --- | --- | --- |
| #1 | Mucosal candidiasis [Title/Abstract] | 242 |
| #2 | Oral candidiasis [Title/Abstract] | 2,670 |
| #3 | Oropharyngeal candidiasis [Title/Abstract] | 827 |
| #4 | Esophageal candidiasis [Title/Abstract] | 625 |
| #5 | #1 OR #2 OR #3 OR #4 | 4,105 |
| #6 | *Candida**[Title/Abstract] | 524,258 |
| #7 | Candidosis [Title/Abstract] | 1,864 |
| #8 | Candidiasis [Title/Abstract] | 18,849 |
| #9 | #6 OR #7 OR #8 | 534,029 |
| #10 | Risk factor*[Title/Abstract] | 822,064 |
| #11 | Predisposing factor*[Title/Abstract] | 21,139 |
| #12 | Risk*[Title/Abstract] | 3,110,883 |
| #13 | #10 OR #11 OR #12 | 3,125,266 |
| #14 | HIV[Title/Abstract] | 366,713 |
| #15 | AIDS[Title/Abstract] | 172,211 |
| #16 | #14 OR #15 | 538,924 |
| #17 | #5 AND #9 AND #13 AND #16 | 195 |

Supplementary TABLE 2. Search strategy and results by Embase database

| **No.** | **Search Terms** | **Results** |
| --- | --- | --- |
| #1 | 'mucosal candidiasis'/exp OR 'mucosal candidiasis' OR (mucosal AND ('candidiasis'/exp OR candidiasis)): ab, kw, ti | 21,226 |
| #2 | oral AND candidiasis: ab, kw, ti | 16,198 |
| #3 | oropharyngeal AND candidiasis: ab, kw, ti | 1,477 |
| #4 | esophageal AND candidiasis: ab, kw, ti | 1,910 |
| #5 | #1 OR #2 OR #3 OR #4 | 30,913 |
| #6 | *candida**: ab, kw, ti | 720,740 |
| #7 | candidosis: ab, kw, ti | 2,515 |
| #8 | candidiasis: ab, kw, ti | 57,050 |
| #9 | #6 OR #7 OR #8 | 748,399 |
| #10 | risk AND factor*: ab, kw, ti | 2,507,796 |
| #11 | predisposing AND factor*: ab, kw, ti | 48,372 |
| #12 | risk*: ab, kw, ti | 5,428,236 |
| #13 | #10 OR #11 OR #12 | 5,452,946 |
| #14 | hiv: ab, kw, ti | 503,886 |
| #15 | aids: ab, kw, ti | 334,460 |
| #16 | #14 OR #15 | 1,974,207 |
| #17 | #5 AND #9 AND #13 AND #16 | 807 |

Supplementary TABLE 3. Search strategy and results by Central database

| **No.** | **Search Terms** | **Results/Hits** |
| --- | --- | --- |
| #1 | (Mucosal candidiasis): ti, ab, kw | 112 |
| #2 | (oral candidiasis): ti, ab, kw | 1,209 |
| #3 | (oropharyngeal candidiasis): ti, ab, kw | 173 |
| #4 | (esophageal candidiasis): ti, ab, kw | 141 |
| #5 | #1 or #2 or #3 or #4 | 1,339 |
| #6 | (*Candida**): ti, ab, kw | 22,229 |
| #7 | (candidosis): ti, ab, kw | 175 |
| #8 | (candidiasis): ti, ab, kw | 2,747 |
| #9 | #6 or #7 or #8 | 23,926 |
| #10 | (Risk factor*): ti, ab, kw | 127,879 |
| #11 | (predisposing factor*): ti, ab, kw | 1,815 |
| #12 | (risk*): ti, ab, kw | 316,998 |
| #13 | #10 or #11 or #12 | 317,771 |
| #14 | (HIV): ti, ab, kw | 32,063 |
| #15 | (AIDS): ti, ab, kw | 28,348 |
| #16 | #14 or #15 | 60,411 |
| #17 | #5 and #9 and #13 and #16 | 32 |

Supplementary TABLE 4. Search strategy and results by Scopus database

| **No.** | **Search Terms** | **Results/Hits** |
| --- | --- | --- |
| #1 | ((TITLE-ABS-KEY(hiv)) OR (TITLE-ABS-KEY(aids))  AND ((TITLE-ABS-KEY(risk AND factor*)) OR (TITLE-ABS-KEY (predisposing AND factor*)) OR (TITLE-ABS-KEY(risk*)))  AND ((TITLE-ABS-KEY(*Candida**)) OR (TITLE-ABS-KEY(candidosis)) OR (TITLE-ABS-KEY(candidiasis))) AND ((TITLE-ABS-KEY (mucosal AND candidiasis)) OR (TITLE-ABS-KEY (oral AND candidiasis)) OR (TITLE-ABS-KEY(oropharyngeal AND candidiasis)) OR (TITLE-ABS-KEY  (esophageal AND candidiasis))) | 846 |
